# Variability and systematic differences in normal, protan, and deutan color naming

**DOI:** 10.3389/fpsyg.2014.01416

**Published:** 2014-12-09

**Authors:** Balázs V. Nagy, Zoltán Németh, Krisztián Samu, György Ábrahám

**Affiliations:** ^1^Vision Laboratory, Institute of Psychology, University of São PauloSão Paulo, Brazil; ^2^Department of Mechatronics, Optics and Engineering Informatics, Budapest University of Technology and EconomicsBudapest, Hungary

**Keywords:** color vision deficiency, color naming, monochromatic visual stimulation, protan and deutan vision, visual psychophysics

## Abstract

The congenital color vision deficient (CVD) generally demonstrates difficulties in color naming tasks. In our study we investigated color naming properties and uncertainties of a relatively large group of red–green CVDs using quasi monochromatic stimuli and seven basic color terms. The results show a large variability in color naming for the CVD when contrasted to normal color vision and similar alterations when comparing protans to deutans. Statistically significant differences were found in specific wavelength ranges between the tested groups. In general, protans and deutans have shown better color naming ability than expected, which suggests the use of non-chromatic visual cues.

## INTRODUCTION

In classical literature a great amount of studies can be found on how the neural system in the eye responds to color stimuli and how chromatic information is processed (Young, 1802; Helmholtz, 1852; Hering, 1905; Hurvich and Jameson, 1957; Kaiser and Boynton, 1996; Wyszecki and Stiles, 2000). Congenital color deficiency has already been studied by several authors (Hurvich and Jameson, 1957; Nathans et al., 1986) and today the genetic background is also well understood (Neitz and Neitz, 2000). The most common type is red–green color deficiency which can be divided into four main subtypes: protanomaly, deuteranomaly, protanopy, and deuteranopy. The former two are the anomalous trichromat types where the trichromatic color vision is preserved but with spectral sensitivity change generally in one of the cone photoreceptors. The latter two are the dichromats which generally means that there are only two functioning cone photoreceptor types (Deeb and Motulsky, 2005).

The functioning of higher order neural color processing mechanisms has also been studied by many research groups. Yet, these issues still raise many questions and require further research work. One of these areas is the color identification and its verbal expression: color naming. This complex process involves all stages in human color perception beginning with the color stimulus up to its association with linguistic terms, i.e., color names. Several researchers have already approached this topic from different points of view, whereas linguistic and geographical distribution of color terms have been broadly studied (Berlin and Kay, 1969; Rosch-Heider, 1972; Kay and MacDaniel, 1978; Kay and Regier, 2003). In their summarizing work on linguistic and geographical aspects of color, Kay and Regier (2003) found eleven basic color terms when comparing different languages and locations. Their corresponding English names are the following: Red, Yellow, Green, Blue, Purple, Brown, Orange, Pink, Black, White, and Gray. However, Saunders and van Brakel (1997) questioned the generality of these 11 terms. Further researchers applied them when testing color naming; taking into account hue, saturation, and brightness at the same time (Boynton and Olson, 1987; le Rochelles and Viénot, 1995; Pitchford and Mullen, 2003). Other studies applied specific samples from color systems (such as the Munsell color chart) when performing color naming tests (Guest and van Laar, 2000; Lillo et al., 2001; Franklin et al., 2005; Bonnardel, 2006; Cole et al., 2006). Moreover, researchers dealt with the spectral dependence of color names (Boynton and Gordon, 1965; Beare and Siegel, 1967; Luria, 1967; Byrne and Hilber, 2003; Troup et al., 2005) including the comparison of normal subjects’ results with those of the color vision deficient (CVD; Scheibner and Boynton, 1968; Smith et al., 1973; Paramei, 1996; Paramei et al., 1998; Diaconu et al., 2010). Most spectral color naming studies restrict the use of color terms to the four basic colors (i.e. blue, green, yellow, and red) with notable exceptions such as Beare and Siegel (1967) who conducted multiple color naming studies applying up to six color names and their compounds. Moreira et al. (2014) have created models based on the CIE u‘v’ chromaticity coordinates and compared them to measurement data. In summary, there is an extensive classical and modern literature on the topic of color naming.

The current study set out to contribute to the existing knowledge on CVD color naming, using the seven basic color terms (the “rainbow colors”) that are monochromatically distinguishable by people with normal color vision. The colors “violet,” “turquoise,” and “orange” were added to the “blue,” “green,” “yellow,” and “red” set (“ibolya,” “türkiz,” “narancs,” “kék,” “zöld,” “sárga,” and “piros” in Hungarian). These were applied within a relatively large group of human subjects, including red–green CVDs and normals. In a previous study (Nagy et al., 2008) using the CVD color naming dataset we have introduced a possible method to categorize CVDs into arbitrary groups. In our current work we intended to provide a more justifiable categorization (comparing with anomaloscope categories) and a more detailed analysis than in previous studies. Mathematical modeling and statistical evaluation was applied to show the spectral differences within and between the tested groups, including color normals, protan and deutan CVDs. Moreover, the relatively large number of CVDs tested enabled us to statistically evaluate the uncertainty of color naming at each test wavelength, which can be characteristic for a given group of CVDs. Hence, it can be used as diagnostic information of the specific color vision deficiency type.

## MATERIALS AND METHODS

### EQUIPMENT

**FIGURE 1:**
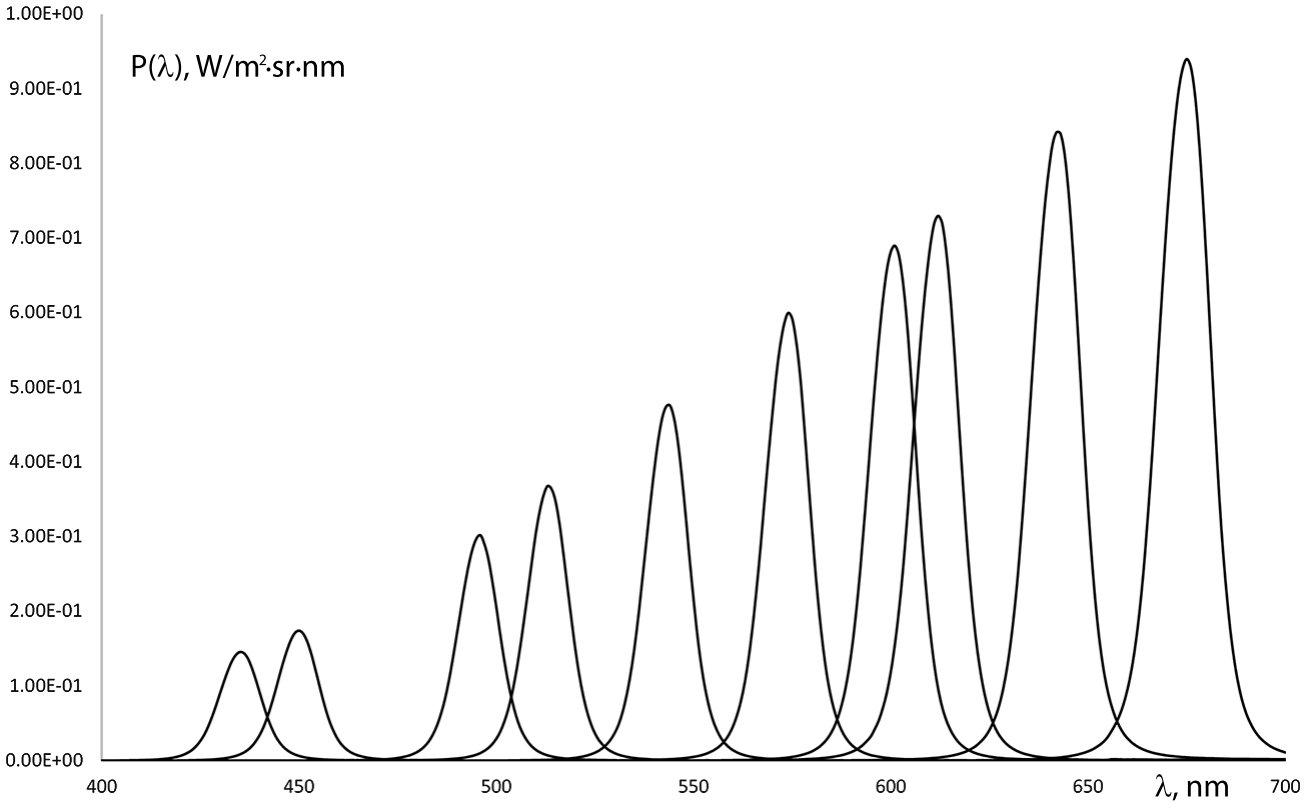
**Spectral radiance distribution variation of some of the stimuli used in the color naming tests**.

A computer controlled test instrument was applied in order to evaluate color naming ability in the human visual spectrum (from 400 to 700 nm). The stimulus consisted of a central 2° field of view target and a 16° surround viewed through the instrument’s eyepiece. The 2° stimulus field targets foveal vision only and eliminates the possibility of rod influence on the results (Nagy and Boynton, 1979; Montag and Boynton, 1987). We used a continuous spectrum constant white background with a spectrally adjustable target stimulus in the center. The target stimulus was realized with a continuous interference filter (IF) of approximately 15 nm FWHM (full width at half maximum). The target luminance, generated by the combination of the light source and the IF filter varied throughout the spectrum within the photopic range (>5 cd/m^2^). Luminance calculations were based on normal color vision, avoiding mesopic luminance levels where significant changes in color naming might occur (Paramei et al., 1998). The radiance of the stimuli was between 1 and 9 W/(m^2^⋅sr) (**Figure 1**). The background luminance had a uniform spectral distribution and was maintained at 120 cd/m^2^ (0.6 W/(m^2^⋅sr)), calculated with the spectral luminous efficiency function of normal color vision.

### TEST SEQUENCE

Prior to the color naming examination, subjects underwent classical color vision tests of Ishihara plates under natural daylight illumination and anomaloscopy using a Heidelberg instrument (Nagel, 1907; Birch, 1993). During the pretesting phase subjects were instructed to adjust the eyepiece of the color naming test instrument. Furthermore, they were able to sweep through the whole spectral range manually, in order to gain a first impression on the expected targets. In real test situations subjects were presented with quasi monochromatic target stimuli in random sequence throughout the visible spectrum with a 10 nm spectral resolution (31 test wavelengths in total). To reduce learning effects and to compensate the non-equiluminance of the target stimuli a spectral shift of approximately 50 nm between consecutive stimuli was applied. Subjects were instructed to name the stimuli using the seven color terms within 2 s to avoid adaptation. If it was necessary, two terms could be ascribed to a stimulus, in which case subjects were asked to indicate the dominant one. The reported color names were recorded. An additional test sequence was applied for the CVD subjects to search for an achromatic neutral target stimulus around 500 nm. During this test the stimulus wavelength was adjusted continuously by the subject and the neutral point’s wavelength was recorded in case of a positive response. Subjects also had to adjust the wavelength at the long wavelength end of the spectrum where the stimulus color faded into the background. Here the threshold wavelength (the so called red-end) has been recorded.

### SUBJECTS

The control group consisted of 31 normal subjects without impairment in color vision (25 men and 6 women), all within the age range of 17–55 with a dominance of the age group 19–23. The CVD group comprised 107 male subjects among whom 22 were diagnosed as protanomals, 26 as protanopes, 30 as deuteranomals, and 29 as deuteranopes. All subjects were of Hungarian nationality and signed a written consent, agreeing to participate in the tests, which adhered the requirements of the institutional regulations.

## RESULTS

Individual subjects were assigned into five categories, i.e., normal, protanomal, protanope, deuteranomal, and deuteranope. Normals were distinguished with the Ishihara plates (Birch, 1993) while CVD groups were assigned when the anomaloscope tests agreed with both the monochromatic neutral point (Wyszecki and Stiles, 2000) and the red end (Kaiser and Boynton, 1996) results. The anomaloscope indicates protanomal, protanope, deuteranomal, and deuteranope categories. Protan results were accepted when indicated by the anomaloscope and if a red-end threshold lower than 700 nm was found. Similarly anope type anomaloscope results were justified by the existence of the neutral point around 500 nm.

**FIGURE 2:**
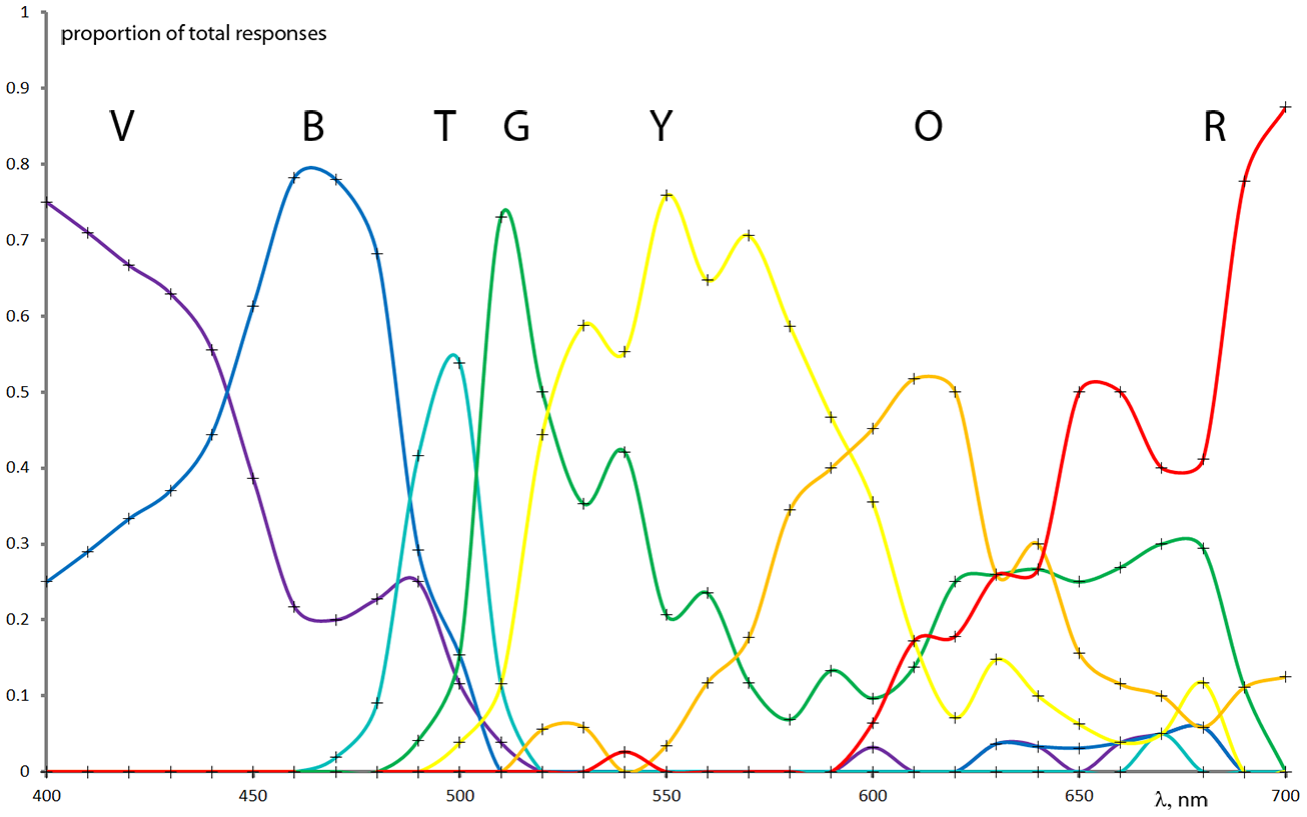
**Interpolation of raw color naming group results normalized to the total number of color names at each wavelength from the deuteranope CVD group (the measured points are indicated with crosses).** Note that the color name distributions can be well approximated with Gaussian types of distributions.

**FIGURE 3:**
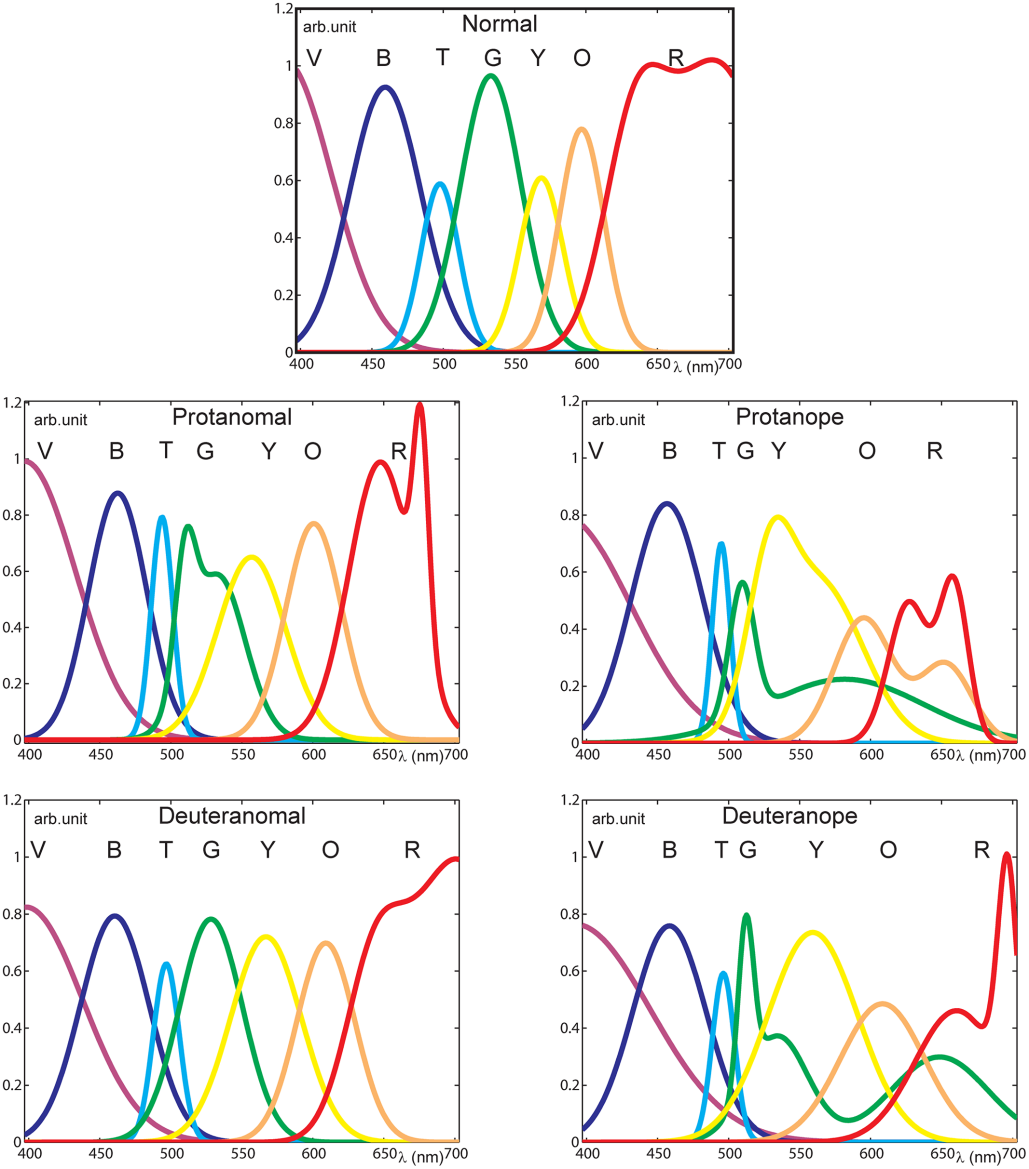
**Normalized pooled color naming results for all categories fitted with Gaussian functions with acceptable goodness of fit.** (Initials of the color names are shown at the peaks of the distributions for the black and white version.) The distributions of color names indicate larger variability in the range from green to red with increasing severity of the deficiency. Note that in protan cases the red distribution ends below 700 nm as their perception in this range is spectrally limited.

The seven color terms’ spectral distributions were normalized at each specific wavelength resulting in the fraction of the total responses for each color term. (See an example of normalized raw data of the deuteranope group in **Figure 2**.) In order to estimate color naming information with higher spectral resolution and to produce specific analysis parameters we applied mathematical models for the measurement results. First and second order Gaussian fits (**Figure 3**) were used to approximate the measured points and to provide analytical functions for each color term in each subject category. The distribution of the results for each color name indicated Gaussian like shapes for the mathematical fitting whereas our measurement data from a relatively large population generally did not show plateaus (Beare and Siegel, 1967) in the color naming spectral distributions (the only exception was the ‘red’ naming of the color normals above 670 nm where the Gaussian fit slightly distorted the plateau without effecting further analysis). Note that some of the distributions could only be approximated with second order Gaussian functions due to the asymmetry of some color name distributions especially in anope cases. The goodness of fit was adjusted for all cases to achieve a sum squared error (SSE) less than 0.10 and a high correlation between the model and the data (*R*^2^ > 0.85).

**FIGURE 4:**
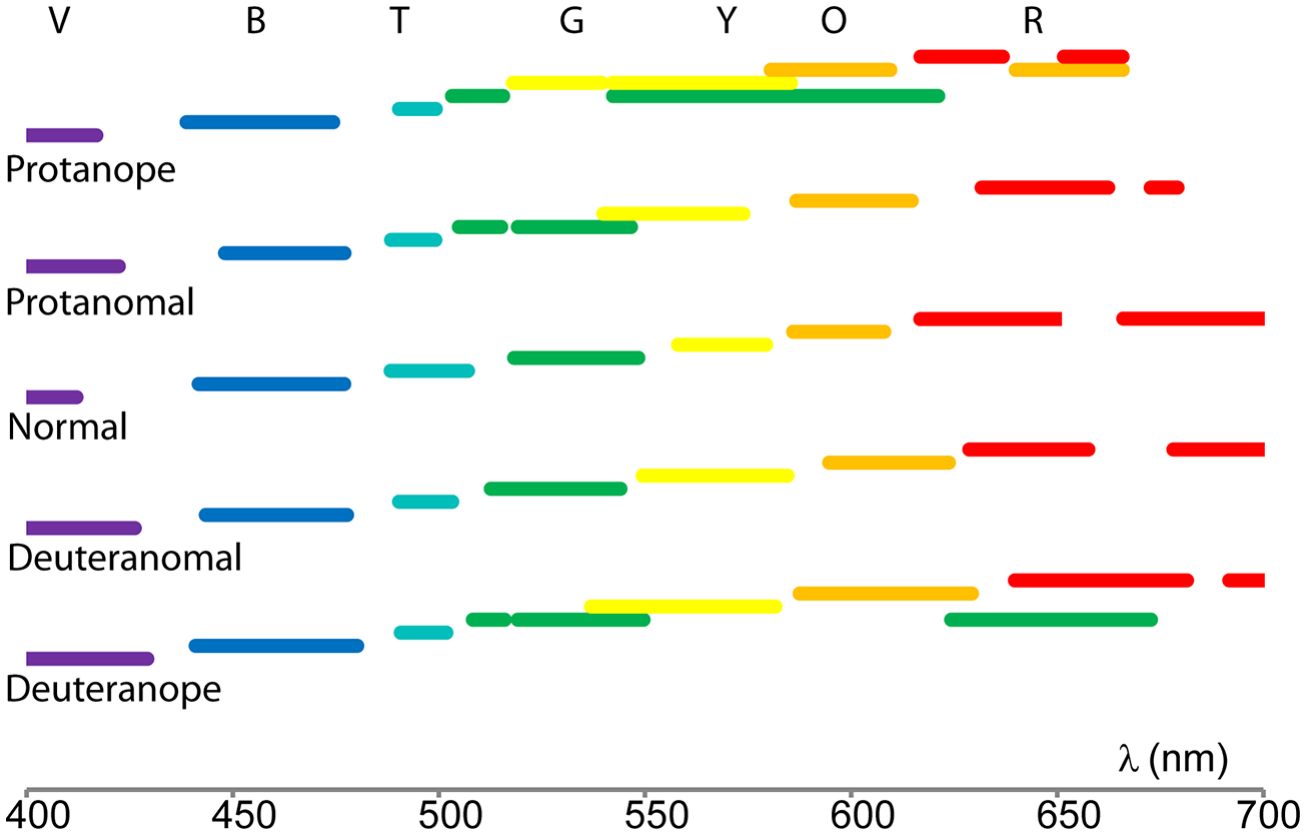
**Color naming ranges of the five tested groups indicated with their colors.** (The color names’ initials are positioned in the middle of the normal ranges.) The overlapping ranges show increase in uncertainty when naming the stimulus color.

Color naming ranges for each color term and subject category were calculated by applying the mathematical fit functions. Results shown in **Figure 3** were processed by using fit parameters (calculating the range of a specific color name using the full width at half maximum centered onto the peak) in order to compare the color naming ranges. **Figure 4** represents how color naming differs in the case of normals and in the four CVD groups. The overlapping ranges show increasing uncertainty of color name use especially in the anope groups.

**FIGURE 5:**
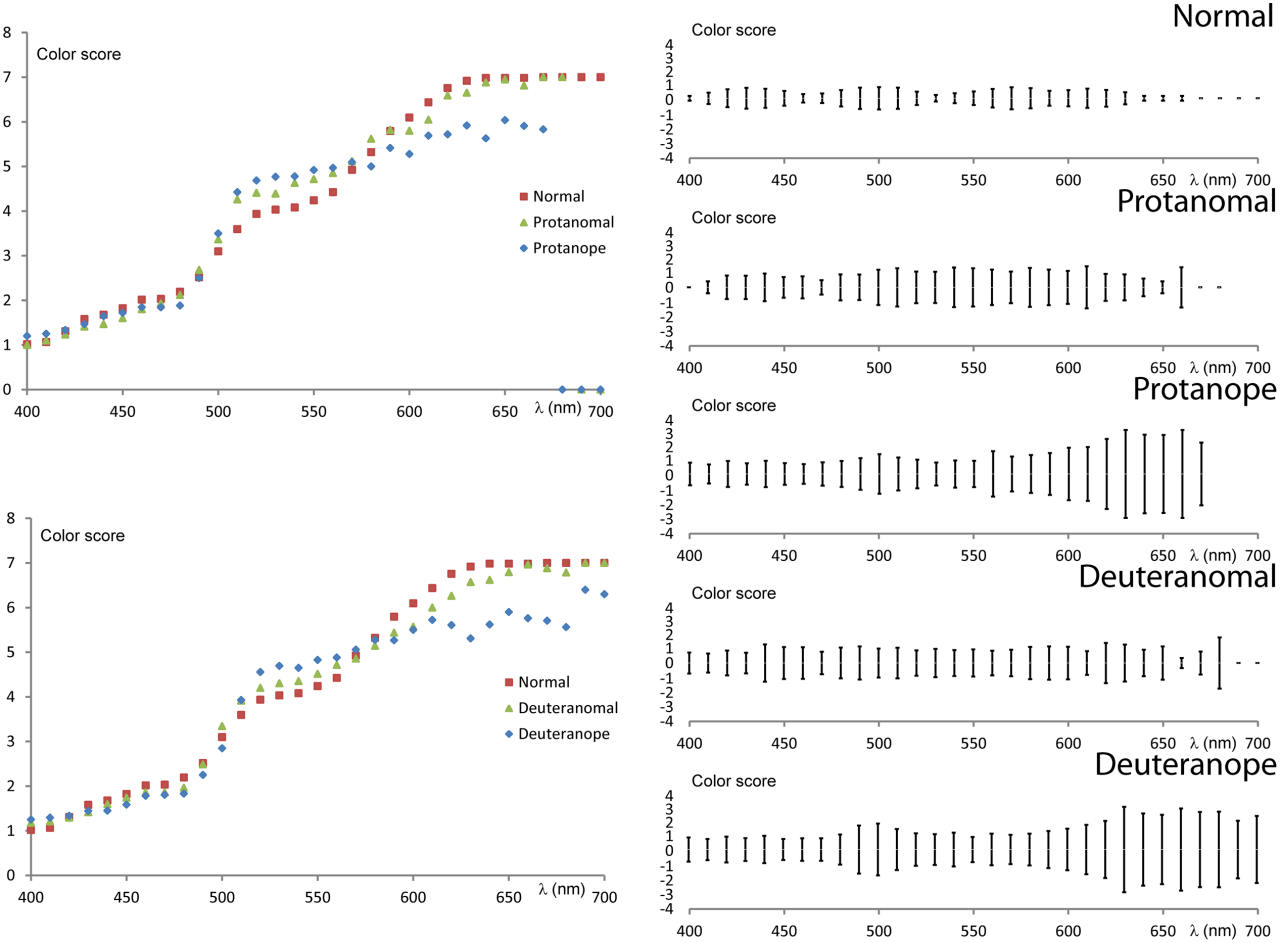
**Numerical evaluation of color naming results.** The two figures on the left show the average scores for each wavelength comparing protans and deutans to normals. (Basic scores are the following: 1 – violet, 2 – blue, 3 – turquoise, 4 – green, 5 – yellow, 6 – orange, 7 – red.) The five figures on the right show the SD of the scores at 95% confidence level.

To analyze the variability of the interpersonal results numerical scores were applied (Nagy et al., 2008) to the ordinal scale of color names ranging from violet to red (1 – violet; 2 – blue; 3 – turquoise/cyan; 4 – green; 5 – yellow; 6 – orange; 7 – red). The numerical scale enabled us to calculate an average score and its SD for each wavelength. These latter values demonstrate the variability of the color naming results in the spectrum. **Figure 5** shows the average scores compared between the groups along with the SD at 95% confidence level.

Color vision deficients generally have larger scores in the “green” range (500 nm < λ < 550 nm) and lower scores in the orange–red range (600 nm < λ) than normals. The difference from normals increases from anomal to anope types. The SD results show a general increase in all groups tested for all wavelengths when compared to normals. However, anopes show larger increase in SD at wavelengths above 600 nm confirming an even higher uncertainty to name colors in this spectral range. Both the average and standard deviation results indicate similarities between the two anomal and the two anope groups, respectively.

**FIGURE 6:**
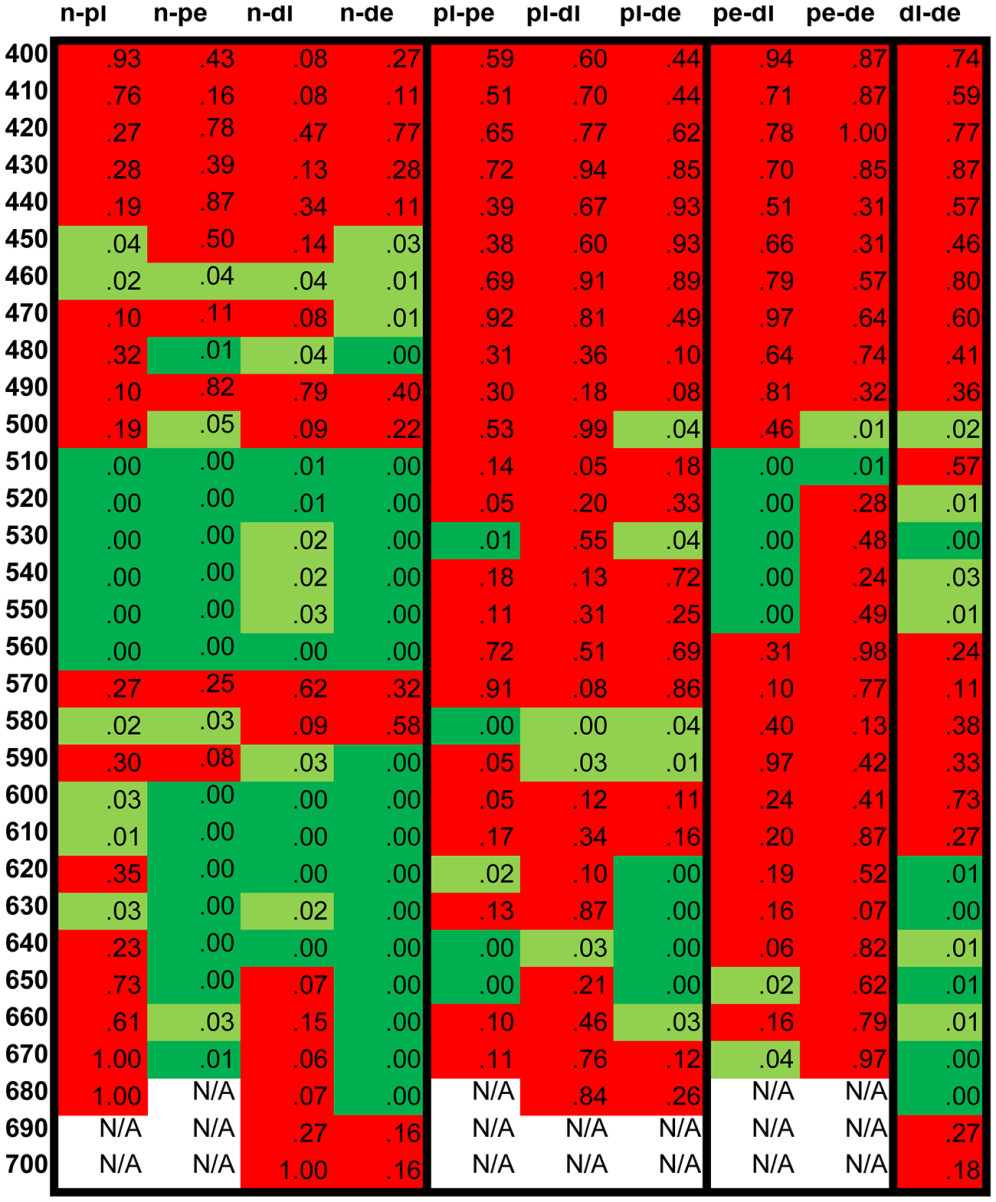
**Statistical analysis of the numerically transformed color naming result.** The numbers in the cells show the *p*-values. The cells are painted light green when the *p*-value is below 0.05 and dark green when it is below 0.01 showing the higher significance level of statistical difference. Red cells indicate *p*-values where no statistically significant differences were detected. n, normal; pl, protanomal; dl, deuteranomal; pe, protanope; de, deuteranope.

To compare the results of the five groups a statistical analysis was carried out, using the non-parametric Mann-Whitney *U* test at each tested wavelength. The table in **Figure 6** displays the results of the analysis showing no statistically significant differences in the shorter wavelength range. The results are significantly different between normals and all groups in the “green” color naming range while in the “orange/red” range the anopes show significant differences at more wavelengths both from the normals and the anomals. Few significant differences have been detected between the two anomal and the two anope groups, which suggest great similarities in color naming between these groups.

## DISCUSSION

Fundamental literature (Smith et al., 1973; Kaiser and Boynton, 1996; Wyszecki and Stiles, 2000) summarizes the basic color identification abilities of CVDs. Our results coincide well with existing literature data (Beare and Siegel, 1967; Luria, 1967; Scheibner and Boynton, 1968; Paramei, 1996) even at different luminance levels. Moreover, they support most of the previous statements, i.e., that CVD subjects have reduced ability in color naming and that they have significantly more yellowish and generally less red experience when identifying hues.

The difference in stimulus brightness is a key issue when considering the test results as this information can be used as a cue for discriminating between colors and also can affect color naming to some extent (Jameson and Hurvich, 1978; Paramei et al., 1998). As the luminous efficiency function varies among normals and CVD groups (Stockman et al., 1993) luminance values will be different for each stimulus for each test subject when using the same stimulus radiance spectral distribution. For this reason the generation of equiluminant stimulation without knowing the exact spectral luminous efficiency function of each subject can be rather complicated and spectral equiluminance based on normal color vision might generate significantly different perceived brightness for CVD subjects at each wavelength. We also need to emphasize the possible, although minor differences in luminance calculations based on the spectral luminous efficiency function – V(λ) – and perceived brightness (Schanda et al., 2002). As the V(λ) function has been determined based on monochromatic tests considering Abney’s Law (Abney, 1913) for polychromatic stimuli, our quasi-monochromatic stimuli might be affected by it resulting in a different perceived brightness from what is predicted by the luminance calculations. These arguments indicate for further research that to equilibrate perceived brightness for color tests one might need an individual brightness calibration for all presented stimuli. Consequently, the stimulation used in our study could not provide the same brightness for all tested groups and individuals; however, the luminance calculated for normal color vision was above the upper mesopic luminance limit. The unequal brightness of the stimulus spectrum can have an effect on the results of color naming; however minor they might be at photopic luminance levels. These should be considered when comparing our results spectrally, thus a better CVD color naming can arise using brightness differences when judging the stimuli at different consecutive wavelengths. Since the same stimulus radiance distribution has been applied for all test subjects, the results demonstrate the between groups spectral comparison of color naming.

Our statistically analyzed results show several differences and also some similarities comparing the five groups tested (including normals). We have found that violet and blue color naming ability seems to be similar for the CVDs when compared to normal. However, the variability of the color name use is increased even for these colors. As expected from protan and deutan types, the larger variation appears at wavelengths above 500 nm. Here we can observe a significant enlargement of the “green,” “yellow,” and “orange” ranges with reduction of the “red” range. Interestingly there are two specific wavelengths where no statistical differences have been detected (490 and 570 nm) when comparing the different CVD groups to normals. These are the regions of the turquoise and the yellow perception peaks. It seems that these two color perception ranges are specifically preserved in the case of red-green color vision deficiency. The numerical transformation describes the variability of the color naming results for the five tested groups. Clearly anopes have more uncertainty when naming color stimuli especially in the spectrum range above 600 nm. However all red–green CVD types have larger uncertainty in color naming for all wavelengths when compared to normals. Another interesting fact deriving from the numerical analysis is that protanope and deuteranope groups have similar color naming when not taking the protans’ spectrally reduced red-end perception into account. Such similarities (or more precisely the lack of statistically significant differences) are observed between protanomals and deuteranomals as well. Thus when considering color naming tasks and applications where the perceptual identification of a color stimulus is necessary (for example in single color identification tasks with no discrimination possibility with other colors) the majority of anomalous trichromats and dichromats (red–green types) could be considered without any specificity to be protans or deutans.

**FIGURE 7:**
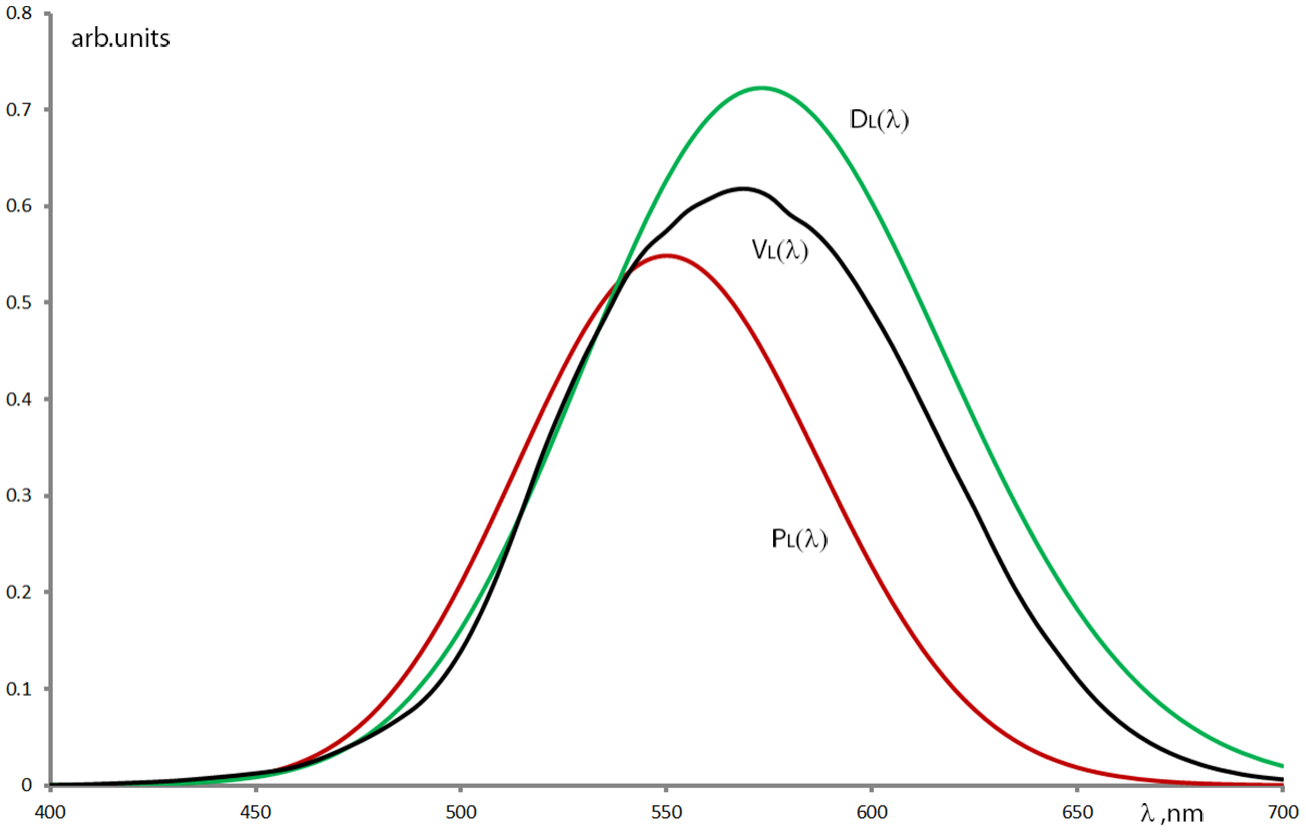
**Relative luminous efficiency functions of protanopes – P_**L**_(λ), deuteranopes – D_**L**_(λ) and normal – V_**L**_(λ) calculated from the data of Stockman et al. (1993) using Gaussian fit onto their measured results and weighted with the spectral radiance distribution of the applied stimuli**.

Another interesting finding arose when looking at the color naming of CVD groups, comprising a relatively large number of subjects. The “green” color name’s range in the pooled anope cases has an additional range at longer wavelengths (around the “orange” wavelength range in the protan case and around the “red” range in the deutan case). This issue of the anopes’ “reappearing green” at the longer wavelengths is still an effect to be discussed. At first instance, it would be considered as an effect of the spectral non-equiluminance of the stimulation. Although as mentioned before, an equiluminant stimulation considering the spectral luminous efficiency of normal color vision would generate different perceived brightness for the CVD groups. Therefore, we used the spectral radiance distribution of our stimulation as a weight function onto the luminous efficiency functions of normals, protans, and deutans (Stockman et al., 1993) to estimate their spectral brightness perception (**Figure 7**). When looking at the color naming ranges in our test results, protans tend to use “green” instead of “orange” while deutans confuse mainly the “red” with the “green.” (Respectively 65 and 51% of the protan and deutan subjects have used the “green” term for naming stimuli at long wavelengths in the “orange” or “red” ranges.) Diaconu et al. (2010) also reported on green naming confusion at 625 nm in the case of protanope subjects, while Bonnardel (2006) showed the appearance of green naming for purple Munsell samples with significant long wavelength spectral content (spectral mixture of the short and long wavelength ends of the visible spectrum) in the case of deutan subjects. However, for “pure” red Munsell samples she did not find such confusions. Bonnardel (2006) provides the hypothesis for the unexpectedly larger diversity of color term use of CVDs: their significantly reduced hue discrimination ability (Kaiser and Boynton, 1996; Wyszecki and Stiles, 2000) in the red–green wavelength region might be enhanced by learning to use other visual cues. The results of the present study seem to support this as even anopes have applied all the seven basic terms allowed in our test with stimuli of spectrally varying brightness. (Nevertheless this latter fact could also be due to a bias in our method as we informed the subjects about the possibility to use all seven color terms.) The hypothesis for such visual cues is that anopes involve the brightness properties (Scheibner and Boynton, 1968; Jameson and Hurvich, 1978; Diaconu et al., 2010) of the different spectral stimuli when judging their chromatic content. In several cases (not only in color naming tests) we have also experienced that anopes (also some anomalous trichromats) tend to describe a chromatic stimulus, first by naming its brightness properties, followed by specifying a hue as if its determination required the preceding brightness judgment (Boynton and Scheibner, 1967; Paramei et al., 1998). Applying the anope color naming results onto the perceived brightness estimation curves in **Figure 7** we can see that the original “green” region (around 510 nm) and the longer wavelength “green” region (around 580 and 640 nm for protans and deutans, respectively) have approximately similar brightness levels. This might be considered as a use of brightness cue when applying quasi monochromatic stimuli for anopes who have reduced chromatic perception for the identification of green and orange/red hues. Obviously the question can arise why this does not happen with other color names especially with a “reappearing orange” at shorter wavelengths? Similarly to the case of CVD color discrimination (Thomson and Trezona, 1951), our hypothesis declares that at the shorter wavelengths the signal of the intact tritos photoreceptor dominates the decision making in color naming tasks even for the anopes.

## Conflict of Interest Statement

The authors declare that the research was conducted in the absence of any commercial or financial relationships that could be construed as a potential conflict of interest.
